# Do farmers value rice varieties tolerant to droughts and floods? Evidence from a discrete choice experiment in Odisha, India

**DOI:** 10.1016/j.wre.2018.03.001

**Published:** 2019-01

**Authors:** Anchal Arora, Sangeeta Bansal, Patrick S. Ward

**Affiliations:** a Shyamlal College, University of Delhi, India; b Centre for International Trade and Development, Jawaharlal Nehru University, New Delhi 110067, India; c Environment and Production Technology Division, International Food Policy Research Institute, USA

**Keywords:** Drought-tolerance, Submergence-tolerance, Rice seeds, Choice experiment, India

## Abstract

Abiotic stresses such as droughts and floods significantly constrain rice production in India. New stress tolerant technologies have the potential to reduce yield variability and help insulate farmers from the risks posed by these hazards. Using discrete choice experiments conducted in rural Odisha, we estimate farmers' valuation for drought-tolerant (DT) and submergence-tolerant (SubT) traits embodied in rice cultivars. Our results demonstrate that farmers in both drought-prone as well as submergence prone regions value reduction in yield variability offered by new, stress-tolerant cultivars, and would generally be willing to pay a significant premium for these traits. While virtually all farmers perceive the threat of drought and are willing to pay for protection against drought risk, only farmers in flood-prone areas would be willing to pay for rice that can withstand being submerged for prolonged periods, suggesting the potential for market segmentation along geographical or ecological lines.

## 1 Introduction

Water stress is an important factor constraining agricultural sector in developing countries as a significant share of farmland is dependent on rainfall for its water needs. About 50% of the total rice farmland in South Asia is rainfed in nature [1]. Variability in rainfall makes these areas susceptible to droughts and floods. The frequent occurrences of submergence and drought are the major causes of crop failure, income volatility and the persistent poverty among the small and marginal rice farmers in South Asia [2]. demonstrates that both drought and submergence significantly affect rice production in Bangladesh.

Rice is a major food crop in India, whose production is significantly constrained by droughts and submergence. About 68% of the total cropping area in India is rainfed [3]. Of the roughly 40 million hectares of harvested rice area in India, only about 60% is irrigated [4] leaving the rest precariously dependent upon rainfall, and hence susceptible to drought. Droughts have obvious consequences in terms of yield reductions, especially if droughts occur during key stages in the rice growth cycle in which plant development is particularly sensitive to water requirements. But droughts may also limit the area under cultivation, such as in the case of delayed monsoon onset. Both of these consequences have potentially severe implications for overall rice production and farm incomes. The value of rice production lost in drought years has been estimated to be as high as 36% of the total value of rice production in eastern India, and the economic costs of droughts to rainfed rice farmers in eastern India are of the order of several hundred million dollars per year [5]. In addition, about 49.8 million hectares of land area (about 15.2% of total geographic area) are prone to floods in India causing a range of losses to human life, property, forests, and crops [6]. Rice seeds that are tolerant to droughts and submergence have a potential to protect farmers from crop losses. The present study aims to evaluate farmers' valuation for drought-and submergence-tolerance characteristics in rice seeds using discrete choice experiment methodology in rural Odisha.

Odisha, located in the eastern part of India, is one of the poorest states of India. Droughts and floods occur frequently in Odisha [7]. The state has been severely affected by five major floods in the last 15 years, including one following the devastating cyclone Paradip in 1999 [8]. Rice is cultivated on an area of 4.45 million hectares. Between 2001 and 2008, around 0.9 million hectares of rice cropped area was exposed to damage by flood and submergence and around 0.8–0.10 million hectares of cultivated rainfed rice was prone to drought stress in 2011 [9].

Rainfed rice production in Odisha is dependent upon the seasonal monsoon, and droughts and floods often arise as a direct consequence of variations in monsoon-season rainfall. But the current understanding of climate change in monsoon-dependent regions (such as India) is plagued with uncertainty, given the complexities of atmospheric circulation and precipitation patterns. The IPCC's special report on Managing the Risks of Extreme Events and Disasters to Advance Climate Change Adaptation (SREX) finds evidence of a general consensus among climate models for an increasing trend in the length of dry spells in much of southern and eastern India (including Odisha) during the 21st century [10]. The current state of scientific knowledge, therefore, suggests a possible future for Odisha in which rice production is increasingly prone to drought and flood-stress. Given that average yields in Odisha are well below the national average, these changes may have significant implications for farm livelihoods.

Substantial scientific effort has gone into breeding water stress tolerant traits into staple crops like rice. In recent years, drought tolerance (DT) globally has received a huge amount of attention from governments and foreign donors. Expenditure on DT research has increased exponentially since 2000 [11].

Under the Stress Tolerant Rice for Africa and South Asia (STRASA) project, rice breeders from the International Rice Research Institute (IRRI) and partner organizations have successfully developed improved, stress-tolerant rice varieties suitable for cultivation throughout South Asia. Of particular note are varieties released for cultivation in eastern India that are tolerant to drought (Sahbhagi dhan) and submergence (Swarna-Sub1). Several recent studies have documented potential benefits from the adoption of stress tolerant rice seeds [12]. estimated that successful development and delivery of DT varieties would produce significant benefits across South Asia, well in excess of the investment necessary to develop the technology. Through a randomized field experiment in Odisha [13], studied the effects of the submergence-tolerant (SubT) rice variety Swarna-Sub1 on rice yields, finding that Swarna-Sub1 had a significant and positive effect on rice yields (relative to non-tolerant varieties) when fields were submerged for as long as 7–14 days. The development and delivery of stress tolerant traits has been seen as a potential avenue through which human livelihoods can be at least partially insulated from the negative impacts of these stresses. But the successful development of these technologies does not imply that the benefits will necessarily be realized; realization of these benefits is contingent upon farmers' actually cultivating the seeds in which these technologies are embodied. Unlike some biotic stress–tolerant seeds (such as insect resistant crops containing the soil bacterium B. thuringiensis—or Bt—in their DNA), the relative benefits of water stress–tolerant cultivars are largely non-monotonic. While the benefits of stress tolerant cultivars are increasing for a certain range of stress severity, they are decreasing for another range of stress severity, and likely zero once the stress level becomes too severe [14]. Additionally, under normal conditions the stress tolerant cultivar may underperform popular non-tolerant varieties. The non-monotonic nature of these relative benefits makes it difficult for farmers to learn about the benefits of the technology, which may hinder its widespread adoption, even where we might objectively expect positive impacts from adoption. For the successful diffusion of new agricultural technologies like DT and SubT seeds, it is important for researchers, breeders, policymakers, and development practitioners to have a deeper understanding of farmers' preferences for crop attributes.

This study aims to fill this gap by studying farmers' preferences for DT and SubT traits. To date there has been relatively little effort to study the potential pathway by which these breeding developments may be translated into actual adoption [15]. is the only other study making such an attempt among Indian rice farmers for DT traits, finding that farmers in drought-prone areas of Bihar, India, largely preferred DT cultivars to status quo. Their study also analyzed the role of behavioral parameters such as risk and loss aversion. In contrast to their study, the present study includes analysis of farmers' preferences for both SubT traits as well as DT traits. In the context of Odisha, it is important to consider preferences for both types of stress-tolerance, since, as noted above, farmers in Odisha are frequently exposed to both drought and flood risks.

We design a discrete choice experiment, and complement the experiment with a separate survey to collect data on socioeconomic characteristics. It is worth mentioning that although a few rice seed varieties tolerant to drought and submergence have been released in India, the seeds are not widely available, further many more are under various stages of development. Seed multiplication takes time and commercial availability on a large scale may take many years. Since, there does not exist any market data for such seeds we employ a stated preference approach namely choice experiment (widely used for economic valuation of non-market goods) to elicit farmers WTP for various attributes in rice seeds.

The next section presents empirical methodology. Section 3 describes data sources, and section 4 presents results. Section 5 discusses the results, and finally Section 6 concludes.

## 2 Empirical methodology

The study relies upon the use of discrete choice experiments to estimate farmers' valuation for different seed traits. In a choice experiment, individuals are presented a series of hypothetical choice scenarios in which they must choose between bundles of different traits, each taking one of a number of pre-specified levels. Through statistical analysis of participants' choices given the alternatives available in each choice scenario, the researcher is able to estimate marginal values (in either utility or monetary terms) for the various attributes embodied in the alternatives. Researchers control the experimental choice environment by providing necessary variation in attribute levels, which may not be present in the historical data. The methodology is particularly useful for getting valuation of products that are yet not on the market, for instance, new technologies that are at a development stage.

There exists a very large literature employing choice experiments in the field of agricultural and resource economics. Some of the studies are provided below. The methodology has been employed to analyze consumer preferences for environmental amenities [[16], [17], [18], [19]], ecosystem services [[20], [21], [22]], food quality attributes [23], and new and improved production technologies [15]. The use of choice experiments in India is relatively rare to date. Most stated choice studies in India have used the traditional contingent valuation approach for the valuation of new agricultural technologies [24,25].

An important condition for the success of choice experiments is that respondents be able to relate to the options that are presented. In this regard, the choice scenarios with which participants are presented should closely resemble real world seed purchasing decisions they face on a regular basis. In an attempt to satisfy this condition, the seed attributes and their respective levels specified in our experiment were carefully chosen so that farmers could relate to them. Prior to designing the choice experiment, we conducted a number of focus group discussions (FGDs) with farmers in Cuttack district of Odisha to identify the attributes that are important to the farmers. We also consulted the existing literature and met with rice scientists at CRRI (Central Rice Research Institute, Cuttack, Odisha) to decide attribute levels. We also tried to ensure that respondents could understand and meaningfully relate to the options. The choice sets and accompanying survey were translated into the local language (Oriya), and the enumerators used the local language in interviewing participants. Visual illustrations were also used for better comprehension of the alternatives.

### 2.1 Attributes and levels

Through the course of the FGDs, farmers identified yield to be the most important trait for selecting a rice variety. Yields, however, are the result of stochastic processes, so characterizing yields, especially in light of these water stresses, requires some finesse. Dalton et al. (2011) used a novel approach to quantify the DT attribute in their study of Kenyan farmers' preferences for DT maize. They described the attribute in terms of not only mean (expected) yield but also the variance of yield distribution under different moisture stress conditions. The approach incorporates non-monotonic nature of the relative benefits of DT variety [15]. modified this approach in studying demand for DT rice among farmers in Bihar, India. We have followed the basic approach of [15] to quantify DT in our study. The DT attribute takes three levels related to different degrees of stochastic dominance over a popular local variety.^2^ In the first of these three levels, the DT first-order stochastically dominates the reference variety: it yields higher under normal conditions, under moderate drought stress conditions, and under severe drought stress conditions. In the second level, the DT second-order stochastically dominates the reference variety: while it does not yield more (nor less) than the reference under normal conditions, it has higher yields under both moderate and severe drought stress conditions. In the third and final level, the DT third-order stochastically dominates the reference variety: the DT yields no more (nor less) than the reference variety under normal and moderate drought stress conditions, but it yields more under severe drought stress. The yield distribution of Sahbhagi Dhan (a recently released DT cultivar) has informed our specification of yield distributions for different levels of stochastic dominance presented in our choice experiment [26]. In order to explain the DT/yield attribute to the respondents, visual illustrations were used in the choice experiment. Each alternative depicted three levels of water stress and associated yield with them under the DT attribute. The water stress was depicted by using pictures of water level in a water tank. A tank that is full up to the rim indicates normal rainfall or no water stress, a half-filled tank indicates mild water stress and a tank with very little water indicates severe water stress. The interviewer explained this to the respondents. Associated with each of the water stress levels, there was a picture depicting the yield ranging from a green cover to a barren land along with a numerical value given in quintals/hectare. Thus, the DT attribute was communicated to the respondents in terms of the yield they could obtain under various water stress conditions.^3^

We include SubT as an attribute with three varying levels, namely, tolerance of 0–5 days, 5–10 days, and 10–15 days of full submergence. Our reference variety, Swarna, can tolerate submergence for 0–5 days, and a recently released SubT rice variety, Swarna-Sub1, can tolerate submergence up to 15 days.^4^

During FGDs farmers also indicated short duration to be an important attribute. This represents another avenue through which breeding research can increase farmers' resilience to droughts. We have included three levels of crop duration—short (less than 120 days), medium (120–135 days) and long (more than 135 days).

We have also included an attribute that distinguishes between seeds that can be stored and reused in the next season and those that cannot.^5^ The seeds that can be stored and reused in the next season have a different impact on intertemporal income and random utility than the seeds that have to be purchased every year, since purchasing seeds that cannot be stored entails incurring seed cost this year as well as farmers' ability to have ready access to seeds in the future periods, as a result of which seed costs have to be incurred every year in future.^6^ Following the literature (e.g., Ward et al.), however, in our choice experiment, we have assumed seed reusability as an attribute whose value gets reflected in the WTP of seeds embodying such attribute. Thus, in a way, our choice experiment captures trade-off between yield enhancing and yield variability reducing attributes in seeds in the current year, and seed reusability in future years.

Finally, since we are ultimately interested in estimating money metric measures for willingness to pay (WTP), we incorporate an additional parameter capturing different prices. To capture the price of inbreds as well as hybrids, and to provide enough variation in seed price levels, we have included six price levels ranging from Rs 15 per kg to Rs 300 per kg in the choice sets.^7^ The attributes and various levels are summarized in Table 1.

**Table 1:** Choice set attributes and respective levels.

Attribute	Levels
Drought-tolerance	“FSD”: Yields 55qtl/ha, 32qtl/ha, 16qtl/ha^a^
	“SSD”: Yields 53qtl/ha, 32qtl/ha, 16qtl/ha^a^
	“TSD”: Yields 53qtl/ha, 22qtl/ha, 16qtl/ha^a^
Submergence-tolerance	0-5 days, 5–10 days, 10–15 days
Duration	Short (90–120 days), Medium (120–135 days), Long (135–165) days.
Seed type	0: Seeds must be purchased every year
	1: Grains which can be storedand used as seed in the next season
Price	Rs 15, Rs 25, Rs 50, Rs 150, Rs 220, Rs 300.

a These figures correspond to yields under normal conditions, moderate drought stress conditions, and extreme drought stress conditions, respectively. A quintal is a unit of mass commonly used in Odisha, equivalent to 100 kg.

**Fig. 1:**
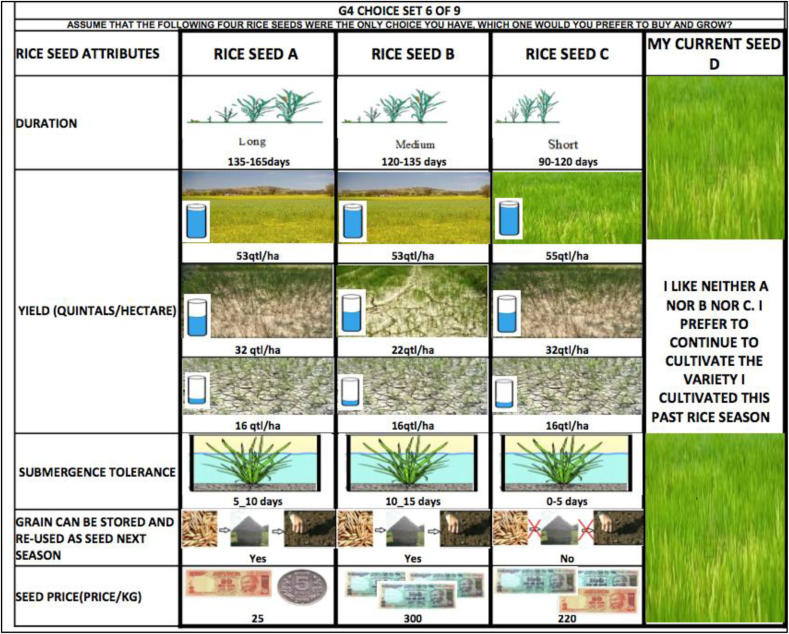
Example of choice set used in discrete choice experiment. Note: While this choice set is in English, the actual choice sets presented to farmers during the discrete choice experiment were translated into the local language (Oriya).

There is a vast literature exploring the many issues pertinent to choice experiment design, and many criteria by which such designs can be evaluated (e.g., [27,28]. Using experimental design methods, the rice seed attributes and their levels were combined into choice sets. We used a blocked fractional factorial design which reduces the number of choice sets given to decision makers, and efficiently estimates the utility parameters. We constructed a D-optimal experimental design based on a main-effects-only linear utility specification with null priors [16,29,30]. This design generated 36 unique choice sets, which were subsequently randomly allocated into four blocks of 9 choice sets each.^8^ Farmers in the sample were randomly allocated to each of these four blocks, with a balanced number of farmers assigned to each of the four blocks. Each choice set contained three alternative hypothetical rice seeds plus a status quo option. The status quo option is the rice seed the farmer used in the past rice season; the information on the rice seed used in the past season and the data on its attributes was collected by the researchers through a primary survey. The status quo for each farmer was then coded in terms of attribute's levels for the econometric analysis. An example of a choice card presented to survey participants is shown in Fig. 1.

Our utility model flows from non-separable agricultural household model where production decisions are guided by consumption and sale [31].^9^ The decision making framework relies on a trait based model of cultivar choice [32]. note that when technology traits exhibit non-pecuniary effects or give rise to nonseparability, it becomes appropriate to use farmers technology adoption decisions within the framework of utility maximizing behavior wherein utility is maximized by choosing a combination of technology traits among a set of feasible alternatives.

### 2.2 Model

Econometric analysis of choice experiment data rests on the framework of random utility theory, which describes discrete choices in a utility maximizing framework [33].^10^ Suppose that individual i faces J alternatives contained in choice set S during choice occasiont. We can define an underlying latent variable $V_{ijt}^{*}$ that denotes the indirect utility associated with individual i’s choosing option j∈Sin choice occasiont. For a fixed budget constraint, random utility maximization implies that individual i will choose alternative j so long as $V_{ijt}^{*}>V_{iqt}^{*}\;\forall q\neq j$. The researcher does not directly observe $V_{ijt}^{*}$ but instead observes the choice made by the respondent, denoted as $V_{ijt}$, where $V_{ijt}=1$ if $V_{ijt}^{*}=\max\left(V_{i1t}^{*},\;V_{i2t}^{*},\;\ldots ,V_{iJt}^{*}\right)$ and 0 otherwise.

Following the standard assumption that utility for an alternative is a linear function of its characteristics,^11^ we can write individuali's indirect utility function as

(1) $$V_{ijt}^{\text{*}}=X_{ijt}^{\text{'}}\beta +\varepsilon_{ijt}\text{,}$$

where$\;X_{ijt}^{'}$ is a vector of attributes for the j^th^ alternative, β is a vector of taste parameters (that is, a vector of weights mapping attribute levels into utility), and $\varepsilon_{ijt}$ is a stochastic component of utility that is independent and identically distributed across individuals and alternative choices, and takes a known distribution. This stochastic component of utility captures unobserved variations in tastes as well as errors in the consumer's perceptions and optimization.

We assume that the random component of utility$\;\varepsilon_{ijt}$, follows a Gumbel distribution. Then, under the assumption that these terms are identically and independently distributed, we can write our expression for the probability of observing alternative jchosen over all other alternatives conditional upon the observed levels of the attribute vector for all alternatives in the choice set S, which gives us the basic conditional logit model and can be estimated using maximum likelihood. The conditional logit framework assumes homogeneous preferences across respondents and independence of irrelevant alternatives [34].

A common method of evaluating preference heterogeneity is estimation of random parameters logit (RPL) models. The RPL is regarded as a highly flexible model that can approximate any random utility model and relaxes the limitations of the traditional conditional logit by allowing random taste variation within a sample according to a specified distribution, and relaxes the assumption of independence from irrelevant alternatives [35]. Following [36]; the probability that individualichooses alternativej from the choice set S in situation t is given by

(2) $$Prob\left(V_{ijt}=1|X_{i1t}^{\text{'}},X_{i2t}^{\text{'}},\ldots ,X_{iJt}^{\text{'}},\text{Ω}\right)=\int \frac{exp\left(X_{ijt}^{\text{'}}\beta_{i}\right)}{\sum_{q=1}^{Q}exp\left(X_{iqt}^{\text{'}}\beta_{i}\right)}f\left(\beta |\text{Ω}\right)d\beta \text{,}$$

where$\;\beta_{i}$ is a vector of (unknown) taste parameters specific to individual i and the matrix Ω defines the parameters characterizing the distribution of the random parameters. The researcher is able to specify the families of these distributions and, where necessary or advantageous, impose restrictions on the distributions. For our purposes, we allow the coefficients corresponding to all attributes except price to vary normally, while the price coefficient is fixed.^12^

Given the utilitarian interpretation of our econometric specification, the vector of parameters $\beta_{i}=\left(\beta_{i1},\;\beta_{i2},\ldots ,\beta_{iK}\right)$ defining tastes and preferences over the K attributes can be interpreted as marginal utilities, and the ratio of two such marginal utilities is simply the marginal rate of substitution of one for the other. If one of the included attributes (say, the K^th^ attribute) is the price of the alternative, then $\beta_{iK}=\beta_{K}$ can be interpreted as the marginal utility of price (or cost), the negative of which is the marginal utility of income (or money). The marginal rate of substitution of money for each of the corresponding attributes—that is, WTP—can be computed as

(3) $$WTP_{ik}=-\frac{\beta_{ik}}{\beta_{K}},\;k\in \left[1,K-1\right]\text{.}$$

The marginal utility (disutility) for favorable (unfavorable) attributes will be positive (negative), indicating that the farmer is willing (unwilling) to substitute an increase in an attribute's expression for money.

## 3 Data sources

The experiments and accompanying survey were conducted with farmers in three different districts of Odisha in June–July 2013. Odisha is one of the largest rice-producing and consuming states in India. The state lies in eastern India and shares its coastline with the Bay of Bengal. The topography of Odisha is such that it contains both low-lying coastal areas, which frequently get flooded for prolonged periods, and rainfed uplands that suffer from moisture stress due to variability in rainfall. The districts included in the study were carefully chosen to include areas that suffer from droughts as well as those prone to prolonged floods.

**Fig. 2:**
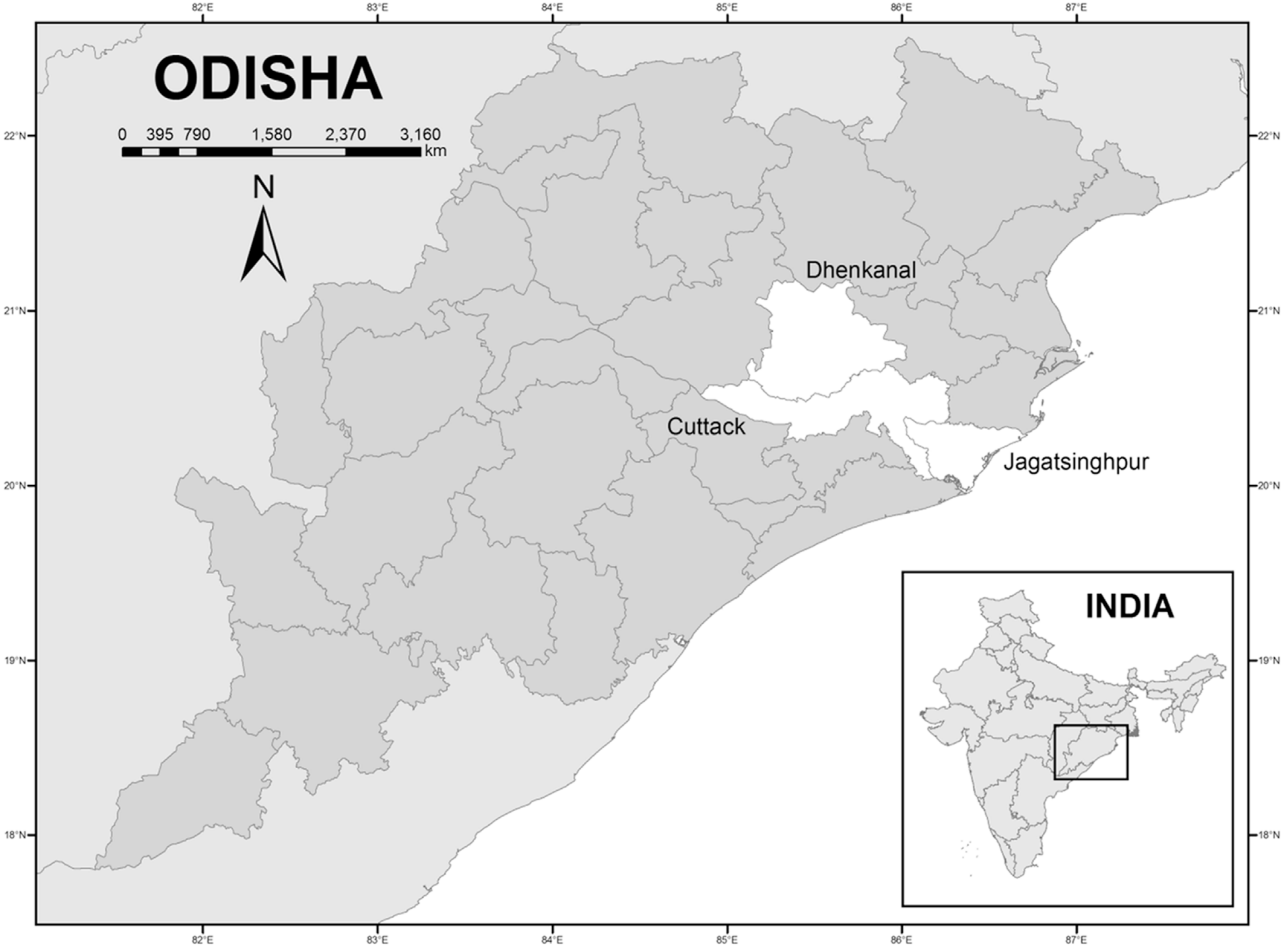
Sample districts.

We used a multi-stage sampling approach to select our survey sample. In the first stage, we identified three adjacent districts in Odisha that are susceptible to drought and/or floods: specifically, we identified the districts Dhenkanal, Cuttack, and Jagatsinghpur (Fig. 2). The district of Dhenkanal has been repeatedly affected by droughts, including in recent years 2002, 2005, 2006, 2008, and 2010. During 2008, it was estimated that more than 70% of total rice area in Dhenkanal was adversely affected due to drought [37]. Dhenkanal is also one of the eight districts in Odisha identified by the government of India for treatment under the Drought-prone Area Programme. Jagatsinghpur district, situated along the Bay of Bengal on the eastern coast of Odisha, is prone to various natural hazards such as floods and cyclones. The district has been severely affected by five major floods in the last 15 years. The third district, Cuttack, demonstrates a great deal of heterogeneity in terms of agro-climatic conditions, with some areas susceptible to droughts and others susceptible to floods.

In the second stage we stratified blocks (sub-district administrative units) within Cuttack and Dhenkanal that were being affected by droughts. The drought-affected blocks drawn for the study are directly proportional to the population of each block as a proportion of total population of all the drought-affected blocks within Cuttack and Dhenkanal. Similarly, we stratified blocks among flood-affected regions of Cuttack and Jagatsinghpur. In this way, we identified four blocks affected by droughts and four blocks affected by floods. Hereafter we will refer to these regions as drought-prone and flood-prone, respectively. We then selected two villages from each of these eight blocks, using probability proportional to size sampling. Finally, we randomly selected 25 rice-growing households in each village from household lists provided by village leaders. The resulting sample consisted of 400 rice-growing households. Descriptive statistics of the sampled farmers are reported in Table 2. While the second column presents the characteristics of the households in the pooled sample, the third and fourth columns present and compare the characteristics of farmers in the drought-prone and flood-prone regions separately. In our sample, as compared with the households in the drought-prone region, the households in flood-prone areas have higher mean annual income, are more educated on average, and have a higher proportion belonging to the general caste. To get a sense of representativeness of our sample, we also report summary statistics of available variables at the level of rural Odisha and rural India in columns 5 and 6. The households in our sample, as compared to households in rural Odisha are more educated on average, have a higher proportion of muslims, a higher proportion of general caste, and a larger proportion employed in agriculture and allied activities.

**Table 2:** Summary statistics of sampled households.

Household Characteristics	Pooled Sample	Drought-prone Blocks	Flood-prone Blocks	Odisha	India
Household size (number of members)	5.24	4.9**	5.6**	4.29	4.67
	(2.01)	(1.70)	(2.23)		
Age of household head	52.88	50.46**	55.3**	47.72	46.62
	(13.68)	(13.33)	(13.60)		
Gender of the household head (proportion)					
Male	96.75%	94.50%	99.00%	89.4%	86.4
Female	3.25%	5.50%	1.00%	10.6%	13.6
Education of household head (proportion)					
Illiterate	17.25%	23.50%	11.00%	40.1%	41.7%
Class 1-5	38.50%	41.00%	36.00%	32.3%	26.4%
Class 6-12	40.25%	31.50%	49.00%	25.3%	28.6%
Bachelor's degree or higher	4.00%	4.00%	4.00%	2.2%	3.2%
Religion (proportion)					
Hindu	95.75%	91.50%	100.00%	98%	84.6%
Muslim	4.25%	8.50%	0.00%	0.8%	11.0%
Caste (proportion)					
General	24.25%	17.00%	31.50%	16.4%	23.2%
Other backward caste (OBC)	40.25%	46.50%	34.00%	37.5%	44.7%
Scheduled caste (SC)	25.00%	16.50%	33.50%	19.2	20.2%
Scheduled tribe (ST)	7.75%	15.00%	0.50%	26.8%	11.9%
Other	2.75%	5.00%	0.50%	–	–
Occupation of household head (proportion)					
Agriculture and allied industries	75.57%	69.35%	82.29%	42.5%	48.1%
Business	5.54%	9.50%	1.56%	15%	11.6%
Daily labor	7.30%	11.00%	3.13%	33*%	32.4*%
Employed (private and government)	4.29%	3.00%	2.60%	–	–
Looking for work or working at home	5.04%	2.00%	7.81%	–	–
Housewife	0.76%	1.00%	0.52%	–	–
Other	1.00%	0.50%	2.00%	9.4%	7.9%
Total annual household income (Rs.)	87,823.74	76,569.96**	99,077.52**		
	(92,183.86)	(95,995.04)	(86,998.93)		
Farming experience (years)	74.95	81.15**	68.75**		
	(1.86)	(2.43)	(2.76)		
Frequency of crop loss due to floods in the last 5 years (proportion)					
1	27.00%	14.50%	39.50%		
2	6.75%	7.00%	6.50%		
3	6.00%		12.00%		
4	0.25%		0.50%		
5	1.75%		3.50%		
Don't know/none	58.25%	78.50%	38.00%		
Frequency of crop loss due to drought in the last 5 years (proportion)					
1	19.50%	34.00%	5.00%		
2	4.00%	7.50%	0.50%		
Don't know/none	76.50%	58.50%	94.50%		
Number of observations	400	200	200		

Notes: Standard errors in parentheses; ** denotes that t statistic for the difference in sample mean of drought prone and submergence prone region is statistically significant. The figures given in columns 5 and 6 are averages for rural Odisha and rural India, respectively. These were calculated from unit level data of schedule number 18.1, Land and Livestock survey, 70th round conducted by NSSO, Government of India.

Table 3 provides information on the distribution of the attributes of rice seeds used in the past rice season. There were no farmers that were cultivating the drought tolerant variety, many were cultivating the reference variety, but there were also many farmers cultivating idiosyncratic landraces. In any event, the yields in alternatives still stochastically dominated farmers' varieties.

**Table 3:** Distribution of the attributes of rice seeds used in the past rice season.

Attribute	Levels	% of farmers
Drought Tolerance	“FSD”: Yields 55qtl/ha, 32qtl/ha, 16qtl/ha^‡^	0
	“SSD”: Yields 53qtl/ha, 32qtl/ha, 16qtl/ha^‡^	0
	“TSD”: Yields 53qtl/ha, 22qtl/ha, 16qtl/ha^‡^	0
Submergence-tolerance	0-5 days	73.22
	5-10 days	17.47
	10-15 days	8.5
Duration	Short (90–120 days)	36.25
	Medium (120–135 days)	37.25
	Long (135–165) days.	26.75
Seed Type	Grains which can be stored and used as seed in the next season	99.25
	Seeds must be purchased every year	0.75

Note: The last column specifies the percentage of farmers reporting cultivating seeds with a specific attribute.

## 4 Estimation and interpretation of results

### 4.1 Econometric results

If all respondents paid due attention to each and every attribute when evaluating the choice tasks, then estimation of the taste parameters could proceed by simply estimating equation (2) by RPL. These estimation results are reported in Table 4.

**Table 4:** Random parameters logit estimates under full attribute attendance model.

	Total Sample			Drought-Prone			Flood-Prone		
	Coef.		Std. Error	Coef.		Std. Error	Coef.		Std. Error
*Random utility parameters*									
Yields 55 qtl/ha, 32 qtl/ha, 16 qtl/ha^a^ (“FSD”)	1.494	***	0.166	1.331	***	0.148	1.519	***	0.169
Yields 53 qtl/ha, 32 qtl/ha, 16 qtl/ha^a^ (“SSD”)	1.519	***	0.164	1.499	***	0.130	1.510	***	0.177
Yields 53 qtl/ha, 22 qtl/ha, 16 qtl/ha^a^ (“TSD”)	1.337	***	0.171	1.258	***	0.147	1.399	***	0.175
SubT - 5–10 days	0.258		0.200	0.349	***	0.101	0.258		0.128
SubT - 10–15 days	0.124		0.199	−0.058		0.119	0.157		0.137
Short Duration	1.208	***	0.367	0.855	***	0.196	1.377	***	0.275
Medium Duration	0.116		0.375	0.194		0.134	0.348		0.201
Seeds cannot be re-used	−0.736	***	0.245	−1.124	***	0.170	−0.595	***	0.145
*Non-random utility parameters*									
Price	−0.365	***	0.048	−0.389	***	0.044	−0.330	***	0.049
*Distribution of random utility parameters*									
Std. Deviation (“FSD”)	1.159	***	0.157	1.083	***	0.138	1.276	***	0.194
Std. Deviation (“SSD”)	0.952	***	0.161	0.685	***	0.162	1.299	***	0.176
Std. Deviation (“TSD”)	1.106	***	0.269	0.874	***	0.151	1.343	***	0.178
Std. Deviation (SubT - 5–10 days)	0.732	***	0.210	0.617	***	0.145	0.964	***	0.194
Std. Deviation (SubT – 10–15 days)	1.032	***	0.203	0.908	***	0.150	1.089	***	0.165
Std. Deviation (Short Duration)	3.114	***	0.418	2.385	***	0.207	4.059	***	0.388
Std. Deviation (Medium Duration)	1.716	***	0.481	1.165	***	0.163	2.478	***	0.264
Std. Deviation (Grain cannot be stored and reused as seed)	1.660	***	0.264	1.775	***	0.171	1.508	***	0.182
N. Replications for simulations	1000			1000			1000		
N. Observations	3600			1800			1800		
N. Parameters	17			17			17		
Log Likelihood	−3843.162			−1972.900			−1837.663		
Pseudo R2	0.229			0.208			0.261		
AIC	7720.325			3979.799			3709.326		
BIC	3912.766			2036.612			1901.375		

Note: * Significant at 10% level; ** Significant at 5% level; *** Significant at 1% level. Presented models were estimated using NLOGIT 5.0.

a These figures correspond to yields under normal conditions, moderate drought stress conditions, and extreme drought stress conditions, respectively. A quintal is a unit of mass commonly used in Odisha, equivalent to 100 kg.

It, however, has been argued in the literature that some respondents may simplify the choice sets by ignoring one or more attributes characterizing the alternatives (attribute nonattendance), which, if not controlled for, results in significantly overestimated measures of WTP [38]. attempted to infer attribute nonattendance from choice experiment data by making use of post-estimation conditioning approaches. If an attribute is serially ignored (i.e., the respondent ignores the attribute in question across all choice tasks), then its contribution to individual utility is zero both within and across choice scenarios, which should be reflected by imposing restrictions on the parameter space for that particular individual. We follow their approach for identifying nonattendance, taking advantage of individual taste coefficients and variation estimated by conditioning the posterior mean marginal utility estimates on observed choices and choice experiment data.

The RPL model allows random taste heterogeneity by allowing marginal utility coefficients to be distributed randomly across respondents in the sample population. The estimated parameters describe the distribution of *β* in the entire sample but do not provide any information on the likely location of a given individual on this distribution. One could obtain more information on individual coefficients by conditioning on the observed choices for specific individuals. This involves making a distinction between the distribution of *β* in the population (unconditional distribution) and the distribution of *β* in the subpopulation of people who faced the same alternatives and made the same choices, yielding the conditional distribution $g\left(\beta_{j}|data_{i},\text{Ω}\right)$. This conditional distribution provides a straightforward method for estimating the expected marginal utility for each subpopulation that responds in a similar fashion when presented the same choice scenario: $E\left[\beta_{ij}\text{|}data_{i}\right]=\int \beta_{j}\cdot g\left(\beta_{j}|data_{i},\text{Ω}\right)d\beta$. For notational simplicity, in what follows we will use the notation $\beta_{ij}$ to indicate the posterior expectation of marginal utility for individual i and attribute j.

To identify attributes ignored by farmers, we compute the coefficient of variation (CV) of the conditional distribution for each attribute level for all the 400 households [38]. A high CV implies that the variation in stated preferences is excessive relative to the mean, which makes the distribution “noisy,” which may imply that the individual is in fact not attending to that particular attribute in his or her decision making process. While the specification of this threshold is admittedly arbitrary, we follow [38] and use a threshold CV of 2 to determine nonattendance. Table 5 summarizes the number of households with a CV greater than or equal to 2 for each attribute. The attributes most often ignored in the pooled sample are medium duration and SubT for 5–10 days, followed by SubT for 10–15 days. For the drought-prone region, the highest number of households ignored the medium duration and SubT for 10–15 days attributes. Again, we can relate these results to statistically insignificant marginal valuations for these two attributes in the RPL estimates for the drought-prone region. Finally, somewhat ironically, the attributes ignored by a large number of households in the flood-prone region are SubT for 5–10 days, grains that cannot be stored and reused, medium duration, and SubT for 10–15 days.

**Table 5:** Summary of ignored attributes.

Attribute Ignored	Number of households		
	Pooled Sample	Drought-prone	Flood-prone
Yields 55qtl/ha, 32qtl/ha, 16qtl/ha^a^ (“FSD”)	23	17	25
Yields 53qtl/ha, 32qtl/ha, 16qtl/ha^a^ (“SSD”)	14	0	26
Yields 53qtl/ha, 22qtl/ha, 16qtl/ha^a^ (“TSD”)	35	1	39
SubT 5–10 days	194	65	92
SubT 10–15 days	160	91	80
Short Duration	65	33	32
Medium Duration	195	96	84
Grains cannot be stored and reused	152	67	86

a These figures correspond to yields under normal conditions, moderate drought stress conditions, and extreme drought stress conditions, respectively. A quintal is a unit of mass commonly used in Odisha, equivalent to 100 kg.^15^

Given that a considerable number of households ignored certain attributes, we re-estimate the RPL model by specifying the coefficient of marginal utility to be zero for ignored attributes in households' utility functions. The results of this constrained RPL model are presented in Table 6, while the sample average WTP for the various attributes is reported in Table 6. RPL results across the two regions are reported in the two right-hand columns of Table 6. Separate estimation was done for each of the regions using 1800 observations for the sample of 200 farmers in each segment. Qualitatively, the results are similar to those of the pooled sample. Marginal utility of attributes associated with DT technology is positive and significant in both the subsamples. While the marginal utility for SubT 5–10 days continues to be positive and highly significant in the drought-prone region, it becomes positive and highly significant for the flood prone region. The marginal utility associated with SubT 10–15 days continues to be insignificant for the drought prone area but becomes positive and significant for the flood-prone region. There is considerably high marginal utility for the short duration attribute in both regions.

**Table 6:** Random parameters logit estimates accounting for attribute nonattendance.

	Total Sample			Drought-prone			Flood-prone		
	Coef.		Std. Error	Coef.		Std. Error	Coef.		Std. Error
*Random utility parameters*									
Yields 55, 32, 16 qtl/ha^a^ (“FSD”)	1.967	***	0.104	1.802	***	0.134	2.545	***	0.166
Yields 53, 32, 16 qtl/ha^a^ (“SSD”)	1.907	***	0.097	1.638	***	0.117	2.478	***	0.164
Yields 53, 22, 16 qtl/ha^a^ (“TSD”)	1.925	***	0.108	1.424	***	0.145	2.731	***	0.171
SubT 5–10 days	0.888	***	0.106	0.987	***	0.106	0.582	***	0.200
SubT 10–15 days	0.143		0.131	−0.258		0.199	0.365	*	0.199
Short Duration	1.700	***	0.250	1.172	***	0.251	1.930	***	0.367
Medium Duration	0.488	*	0.257	0.638	***	0.245	0.925	**	0.375
Seeds cannot be reused	−1.900	***	0.173	−2.274	***	0.258	−1.581	***	0.245
*Nonrandom utility parameters*									
Price	−0.402	***	0.032	−0.398	***	0.044	−0.426	***	0.048
*Distribution of random utility parameters*									
Std. Deviation (“FSD”)	0.883	***	0.106	0.792	***	0.128	0.786	***	0.157
Std. Deviation (“SSD”)	0.696	***	0.119	0.458	**	0.195	0.740	***	0.161
Std. Deviation (“TSD”)	0.780	***	0.134	0.910	***	0.169	0.355		0.269
Std. Deviation (SubT: 5–10 days)	0.861	***	0.152	0.012		0.217	1.470	***	0.210
Std. Deviation (SubT: 10–15 days)	1.522	***	0.151	1.402	***	0.199	1.576	***	0.203
Std. Deviation (Short Duration)	3.562	***	0.258	2.780	***	0.277	4.435	***	0.418
Std. Deviation (Medium Duration)	3.060	***	0.326	2.106	***	0.254	3.627	***	0.481
Std. Deviation (Grain cannot be stored and reused as seed)	2.138	***	0.179	2.163	***	0.271	1.966	***	0.264
N. Replications for simulated probabilities	1000			1000			1000		
N. Observations	3600			1800			1800		
N. Parameters	17			17			17		
Log Likelihood	−3383.308			−1763.921			−1536.256		
Pseudo R2	0.321			0.291			0.382		
AIC	6800.615			3561.842			3106.512		
BIC	3452.911			1827.633			1599.968		

Note: * Significant at 10% level; ** Significant at 5% level; *** Significant at 1% level. Presented models were estimated using NLOGIT 5.0.

a These figures correspond to yields under normal conditions, moderate drought stress conditions, and extreme drought stress conditions, respectively. A quintal is a unit of mass commonly used in Odisha, equivalent to 100 kg.

Although marginal utilities cannot be directly compared across the two groups due to potential differences in scale effect, WTP can be, since scale effects are eliminated. It is worth noting that the valuations are consistently higher for all the attributes in the flood-prone region as compared with the drought-prone region, with the ironic sole exception of SubT for 5–10 days. The difference in valuations is remarkably large for the short duration attribute and for having to purchase new seed every year. As we will later show, this result will have a bearing on the WTP estimates in the two regions.

### 4.2 Willingness to pay

Table 7 reports willingness to pay estimates. On average, farmers have a positive and statistically significant WTP for the productivity-increasing as well as risk-reducing attributes associated with DT technology. In the drought-prone sample, the WTP for the first-order stochastic dominant (FSD) yield distribution is larger than the WTP for the second-order stochastic dominant (SSD) yield distribution, which is in turn higher than the WTP for the third-order stochastic dominant (TSD) yield distribution.^13^ In the flood-prone sample, however, the WTP for the third-order stochastically dominant (TSD) yield distribution is actually higher than the WTP for the other two distributions. Farmers in the flood-prone areas, therefore, are willing to pay a higher premium for seeds that reduce their exposure to severe droughts, while not necessarily demanding an increase in expected yields (yields under normal conditions) or less variability. Farmers in the flood-prone sample have, on average, a positive WTP for long-term submergence tolerance (though the average WTP is only statistically significant at the 10% level). In drought prone areas, the coefficient for 5–10 days submergence tolerance is positive and significant, however, the coefficient of submergence tolerance 10–15 days is negative but insignificant. The latter may appear as an unexpected finding, as one would expect a positive and a larger coefficient for the 10–15 days submergence tolerance. One possible explanation could be because that those residing in drought-prone areas may not perceive susceptibility to floods of such a prolonged duration as to not justify a large valuation. In other words, they may not derive any utility from a trait from which they do not expect to ever reap a benefit.

**Table 7:** Sample average WTP for seed attributes in Indian Rupees.

	Drought-prone sample			Flood-prone sample		
	Lower 2.5%	Mean	Upper 2.5%	Lower 2.5%	Mean	Upper 2.5%
Yields 55, 32, 16 qtl/ha (“FSD”)	369.577	452.311	569.669	491.564	597.744	747.801
Yields 53, 32, 16 qtl/ha (“SSD”)	336.030	410.842	518.057	477.140	581.031	729.543
Yields 53, 22, 16 qtl/ha (“TSD”)	281.480	356.732	456.289	533.340	640.856	794.081
Submergence-tolerance (5–10 days)	184.072	247.291	332.421	44.200	136.030	241.626
Submergence-tolerance (10–15 days)	−170.038	−64.671	34.230	−5.539	86.487	185.366
Short Duration (less than 120 days)	169.479	294.968	446.087	275.693	454.860	673.776
Medium duration (120–135 days)	37.797	160.877	297.277	46.839	218.891	412.372
Seed must be purchased every year	−773.518	−571.790	−418.611	−527.963	−370.202	−248.936

Note: Confidence intervals derived using parametric bootstrap procedure introduced in Ref. [43] based on 10,000 random draws from a multivariate normal distribution with means and variance-covariance matrix of the estimated model parameters. A quintal is a unit of mass commonly used in Odisha, equivalent to 100 kg.

Not surprisingly, we find positive, significant, and considerably high marginal utility for the short duration attribute. There are various possible reasons for this. First, short duration provides a means of escaping the abiotic stress—drought or flood. If droughts arise due to rainfall deficiencies at the two ends of the growing season (that is, due to late monsoon onset or early monsoon cessation), short duration allows farmers to either delay transplanting (in the case of delayed monsoon onset) or harvest a fully mature crop without creeping into *rabi* season land preparation. Second, short duration may allow farmers to grow another short duration crop between the *kharif* and *rabi* crops. These crops are typically higher-value horticultural crops, which provide increased farm incomes as well as income diversification. Third, short duration rice may provide farmers with a window of time in which to pursue nonagricultural income. Short duration varieties can also help in alleviating the hunger period, the pre-harvesting period when typically food availability is low and prices are high. If some crops mature earlier, they can provide food in this period.

Our results suggest that if seeds that produce grains that cannot be stored and reused as seed in the next season have no yield advantage then farmers would demand a high discount to cultivate them. However, the hybrids available in markets are often more expensive than the open pollinated varieties. This may be because hybrids usually have high yield potential embodied in them, and thus the price of hybrids that we see in actual markets reflects valuation for yield improvement and other benefits conferred in them discounted by the lack of seed reusability trait.^14^

### 4.3 Demand curves

We can use the estimated coefficients from the RPL model to calculate aggregate demand for new seeds with specified attributes using the sample enumeration technique. The probability of choosing different alternatives is predicted for each individual by using the estimated coefficients in conjunction with equation (2). The predicted share of the sample choosing different alternatives is then obtained by summing over these probabilities. The demand for alternative j,denoted by D(j), is given by

(4) $$D\left(j\right)=\sum_{i=1}^{n}Prob\left(V_{ijt}=1|X_{j};\;\hat{\beta }\;\right)\text{,}$$

Where $\hat{\beta }$ denotes coefficients estimated from the RPL model and n denotes the number of individuals in the sample. To get a share, the demand is divided by the number of individuals in the sample. Different demand functions are generated by varying the vector of observable attributes $X_{j}$, keeping the parameter estimates $\hat{\beta }$ unchanged.

**Fig. 3:**
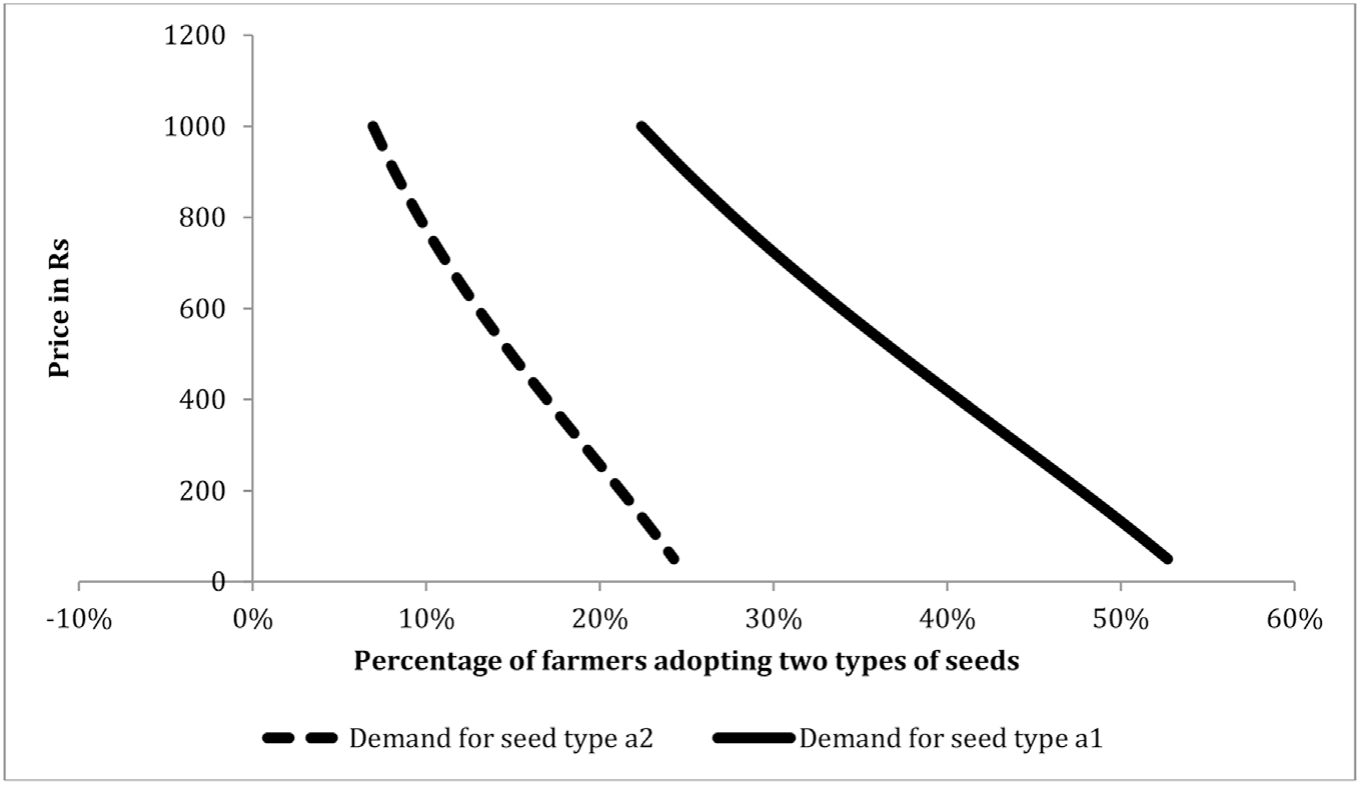
Simulated demand for two seed types, $a_{1}anda_{2},\;$in the pooled sample:$a_{1}$is a short duration, DT inbred variety, while $a_{2}$ is medium duration, subT (10–15 days) inbred variety.

**Fig. 4:**
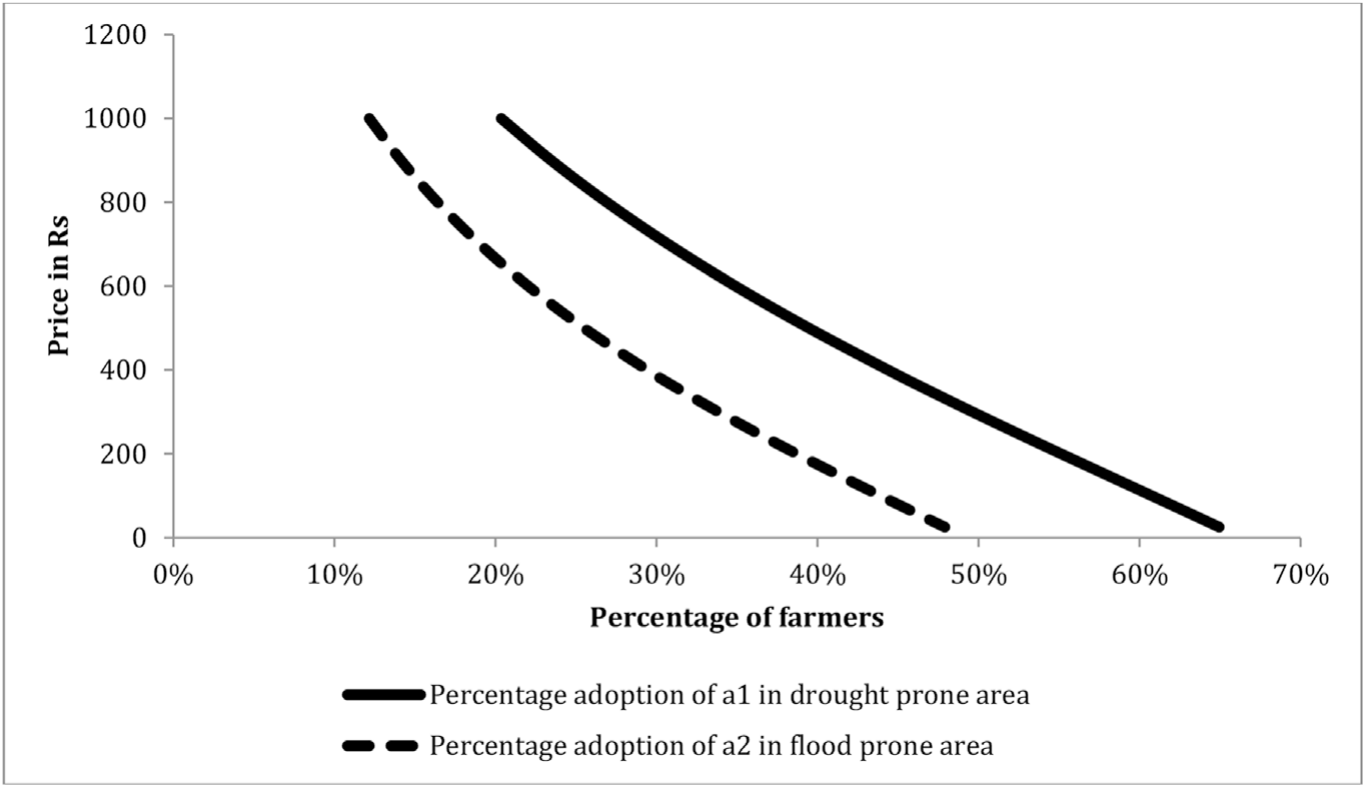
Simulated demand for two seed types $a_{1}anda_{2}$in the drought-prone and flood-prone regions, respectively:$a_{1}$is a short duration, DT inbred variety, and $a_{2}$ is a medium duration, subT (10–15 days) inbred variety.

Suppose, for example, we are considering introduction of two alternative new seeds, called $a_{1}\;\text{and}\;a_{2}.\;\;$Seed $a_{1}\;$has properties similar to Sahbhagi dhan: it is a short duration inbred variety with yields along the lines of the FSD distribution introduced earlier. Seed $a_{2}$ has properties similar to Swarna-Sub1: it is a medium duration inbred rice variety that can tolerate being submerged for up to 15 days. Fig. 3 illustrates the predicted demand vis-à-vis status quo if one of these seeds was introduced in the pooled sample as the price of the new seed varies from Rs 50 per kg to Rs 1000 per kg. The continuous curve and the dashed curve depict demand for $a_{1}\text{and}\;a_{2},\;\text{respectively}$, and each curve depicts the percentage of farmers who would choose the new seed at various prices. As expected, the demand curves slope downward indicating that, as the price increases, a smaller proportion of farmers would be likely to purchase the seed. Clearly the demand for seed type $a_{1}\;$ is higher than that of seed type $a_{2}.\;$Fig. 4 illustrates the demand curves for $a_{1}\;\text{and}\;a_{2}$ in drought prone and flood prone regions, respectively: the continuous curve represents percentage of farmers that would adopt $a_{1}\;$vis-à-vis the status quo in the drought prone region, while the dashed curve depicts the percentage of farmers that would adopt $a_{2}$vis-à-vis the status quo in flood prone regions. Again the figure illustrates that the demand for the seed having subT attribute is low even in the flood-prone region.

### 4.4 Socio-economic characteristics

Using various versions of RPL model, we tried to explore the reasons for heterogeneous preferences of farmers by interacting rice seed attributes with farmers socio-economic characteristics such as income, caste etc. We find that different groups of farmers have different valuation for these new and improved technologies. More specifically, while the high income farmers have a higher valuation for yield enhancing (FSD), submergence tolerance, short duration attributes, the lower income farmers highly value SSD, TSD and seed reusability attributes. Further, the non-scheduled groups have a higher valuation for almost all rice seed attributes as compared to scheduled caste groups. One of the limitations while running separate RPL regressions interacting with income and caste is that in each regression we have potentially confounded effects, i.e., we cannot isolate the effects of income from that of caste in each regression because scheduled caste households are likely to belong to the lower income group.

## 5 Discussion

The results suggest that, on average, farmers in the pooled sample are willing to pay a significant premium for DT technologies. The different valuations for the three yield distributions illustrated here suggest that, while farmers value reductions in yield variability and protection against downside risk, they may not be willing to sacrifice higher expected yields to attain these. This result is close to [15] and [39] in their analysis of farmers' preferences for different yield distributions in rice seeds in Bihar, India, and maize seeds in Kenya, respectively. Farmers also highly value short duration rice, as well as being able to store grain and use it as seed in subsequent seasons. Qualitatively, the results are similar for the subsamples of drought-prone and flood-prone regions; however, the WTP for most attributes is considerably higher in the flood-prone region than in the drought-prone region, which reflects the socioeconomic differences in households from the two regions. Households in flood-prone areas, on average, have higher mean incomes, are more educated, and are more likely to belong to the general caste.

It should be duly noted that, while we made efforts to ensure the respondents treated the choice scenarios as they would real-world purchasing decisions, these WTP estimates are likely biased upward due to the hypothetical nature of the choice exercise. While many observers are generally of the opinion that farmers appreciate improvements in seed technologies and would be willing to pay a premium for these improvements, the marginal WTP estimates in the Odisha samples seem perhaps unreasonably high. Since we have no alternative but to assume that the observed choices are reflective of farmers' true preferences given the options they are confronted with, and that the valuations are at least consistent with preference orderings, if not of the exact monetary scale that farmers would actually pay.

A robust result from our study is that the valuation for the DT technology is significantly higher than the valuation for the SubT technology. Our characterization of DT and SubT traits may have a bearing on this. While we have characterized DT in terms of yield distributions under different moisture stress conditions, SubT is described in terms of number of days of submergence that crops can tolerate. The cognitive burden associated with the DT attribute is probably greater than the one associated with the SubT attribute. These differences in characterization might have influenced the choice process.

Another robust result from the study is high valuation for the short duration rice. By cultivating a short duration variety, farmers can escape droughts and floods; however, our results suggest that farmers value the short duration variety even beyond its ability to escape abiotic stresses. In addition, farmers cultivating short duration rice are able to supplement their income by either cultivating a short duration vegetable crop after harvesting their *kharif* rice or by taking advantage of non-agricultural opportunities after the *kharif* harvest.

Some studies conducted in Africa and Asia have investigated adoption and farmers' valuation for improved seeds that have DT attribute [40]. survey farm households in six African countries to measure DT maize adoption rates and their determinants. They find considerable inter-country variation in farmer adoption of DT maize ranging from 9% in Zimbabwe to 61% in Malawi. The major barriers to adoption include unavailability of improved seed, lack of awareness and understanding about its benefits, lack of resources, and high seed price [39]. use a choice experiment to elicit WTP for DT attribute in maize seeds in eastern Kenya using a latent class model. They find the WTP to be highly heterogeneous, and the WTP of the segment having the highest WTP was consistent with observed market price for hybrid seeds [41]. investigate low adoption of improved Sorghum varieties in Ethiopia. Their results suggest that risk-factors coupled with access to markets and social capital drive farmers' decision to adopt improved varieties of Sorghum. In a discrete choice experiment study conducted in Bangladesh [42], find that farmers are not willing to adopt a DT rice variety without substantial financial incentives such as subsidies. However, the marginal utility of DT rice increases if the seed is bundled with insurance.

## 6 Conclusion

Abiotic stresses such as droughts and floods lead to significant income and consumption losses for rice-growing farmers in India. Rice seed varieties that have better tolerance to moisture stress conditions and submergence have the potential to protect farmers' livelihoods. The objective of this study was to estimate farmers' valuations for drought and submergence-tolerance characteristics in rice seeds using a discrete choice experiment methodology for 400 rice-growing farmers in three districts of Odisha. We find considerable heterogeneity in the farmers' valuations for various attributes in rice seeds, and thus we report results from an RPL model. In the reported results we have accounted for attribute nonattendance.

We find that farmers in both drought-prone and submergence-prone regions are generally willing to pay a significant premium for a reduction in yield variability offered by the new rice varieties. However, only farmers in flood-prone areas are willing to pay for rice that can withstand being submerged for prolonged periods. The valuation for the DT technology is significantly higher than the valuation for the SubT technology. Farmers also highly value short duration rice as well as seed reusability. The valuation for most traits is considerably higher in the flood-prone region.

The results from our study are useful for researchers developing these new technologies in determining the traits they should focus on. They are also useful in guiding public and private sector investment in the development and delivery of such technologies. Our results suggest the existence of naturally occurring market segments. In particular, the result that farmers in both drought-prone and flood-prone areas are willing to pay for seed technologies that provide protection against droughts suggests that all farmers—even those not residing in designated drought-prone areas—perceive susceptibility to droughts. This may be a result specific to Odisha, in which many areas have historically endured prolonged dry spells. Furthermore, much of Odisha has precariously little irrigation infrastructure with which to buffer production during these dry spells.

A different story emerges altogether when we consider potential markets for SubT varieties such as Swarna-Sub1. Farmers in drought-prone areas are very clearly not willing to pay for protection from prolonged floods, and so would not be a viable market place for such products. There does appear to be potential demand for SubT products in flood-prone areas, but the marginal WTP for protection against prolonged submergence is small in comparison with the marginal WTP for other traits, such as shorter duration. This perhaps suggests that while farmers in flood-prone areas might be aware that floods are a threat to their production, floods of such a prolonged duration are sufficiently rare as to not justify a large valuation. While there is a positive valuation, this is perhaps a cautionary note about the potential success of marketing a product like Swarna-Sub1.

Our estimation results also depict considerable heterogeneity in the preferences of farmers for attributes like short duration, medium duration, seed reusability, and yield variability. Future research can explore the role of socioeconomic and behavioral factors causing this heterogeneity, and of policy interventions that ensure that new technologies are accessible to all sections of society.

## Funding source

This work was funded by the United States Agency for International Development (USAID) and the Bill and Melinda Gates Foundation under the Cereal Systems Initiative for South Asia (CSISA) (OPP1052535).

## Footnotes

2 Swarna, a popular rice variety in Odisha that gives a yield of 53 quintals per hectare (qtl/ha) under normal conditions, 22 qtl/ha under moderate drought stress, and 6qtl/ha under severe drought stress [26], has been considered as the reference variety.

3 Table 5 shows that less than 10% of the respondents ignored yield attribute suggesting that the respondents were able to comprehend the attribute.

4 In a series of field trials [44], found Swarna-Sub1 to withstand submergence for up to 17 days. Given likely differences in conditions between agronomic field trial plots and farmer fields, we have assumed a slightly reduced degree of tolerance.

5 Most of the benefits that arise as a result of the hybridization process (such as increased productivity and uniformity) are fully expressed in first generation seeds but decline dramatically in subsequent generations. Therefore, farmers must purchase new hybrid seeds every year in order to continually realize these benefits.

6 We thank the anonymous referee for pointing this out to us.

7 The market price of hybrid rice is varying between Rs 200 and Rs 250 per kg in India [45].

8 D-optimal designs minimize the D-error of the design, which is computed as the weighted determinant of the variance-covariance matrix of the design, where the weight is an exponential weight equal to the reciprocal of the number of parameters to be estimated. The D-efficiency and D error reported for our design are 5.1181 and 0.1954 respectively.

9 In our sample, approximately 34% of the total rice harvested during Kharif and Rabi season 2012 is sold for commercial purposes, 36% is used for self consumption and the remaining 30% is kept as seed or used for other purposes such as gift, wages, etc.

10 Utility maximization is one of a number of possible paradigms by which we can classify farmer behavior. Other paradigms include regret minimization, lexicographic preferences, and so on. In what follows, we will follow the assumption of [46] and [33] and assume that the process of utility maximization necessarily incorporates errors due to imperfect perception and optimization, as well as the inability of the econometrician to exactly measure all of the relevant variables [47]. The utility function will therefore be a random function with both a deterministic component and a stochastic component.

11 This implies that marginal utility is strictly monotonic in traits and gives rise to a corner solution in which only one product is purchased [32].

12 Since the WTP for an attribute is given by the ratio of the attribute coefficient to the price coefficient, the WTP from a mixed logit model is given by the ratio of two randomly distributed terms. Depending on the choice of distributions for the coefficients this can lead to WTP distributions which are heavily skewed and that may not even have defined moments. A common approach to dealing with this potential problem is to specify the price coefficient to be fixed. This is a convenient assumption as in this case the distribution of the willingness to pay for an attribute is simply the distribution of the attribute coefficient scaled by the fixed price coefficient [48]).

13 It should be noted, however, that the sample average WTP estimates are not statistically distinct from one another.

14 While hybrids largely confer higher and more uniform yields at lower seeding rates than varieties (i.e., a lower quantity of seed is required to cultivate a given area), hybrid seeds must be purchased anew every year for the benefits to be observed.

15 Under each partition, the parameters are estimated separately, so it is possible to reach different determinations about ANA based on the particular sub-sample estimates, and therefore, the numbers from the two sub-samples may not add to the number from the pooled sample.
